# Restriction of diverse retroviruses by SAMHD1

**DOI:** 10.1186/1742-4690-10-26

**Published:** 2013-03-05

**Authors:** Thomas Gramberg, Tanja Kahle, Nicolin Bloch, Sabine Wittmann, Erik Müllers, Waaqo Daddacha, Henning Hofmann, Baek Kim, Dirk Lindemann, Nathaniel R Landau

**Affiliations:** 1 Microbiology Department, New York University School of Medicine, New York, USA; 2 Virologisches Institut, Klinische und Molekulare Virologie, Universität Erlangen-Nürnberg, Erlangen, Germany; 3 Institut für Virologie, Medizinische Fakultät Carl Gustav Carus, Technische Universität Dresden, Dresden, Germany; 4 Department of Microbiology and Immunology, University of Rochester Medical Center, Rochester, New York, USA

**Keywords:** HIV, SAMHD1, Macrophages, Vpx, Dendritic cells, Accessory proteins

## Abstract

**Background:**

SAMHD1 is a triphosphohydrolase that restricts the replication of HIV-1 and SIV in myeloid cells. In macrophages and dendritic cells, SAMHD1 restricts virus replication by diminishing the deoxynucleotide triphosphate pool to a level below that which supports lentiviral reverse transcription. HIV-2 and related SIVs encode the accessory protein Vpx to induce the proteasomal degradation of SAMHD1 following virus entry. While SAMHD1 has been shown to restrict HIV-1 and SIV, the breadth of its restriction is not known and whether other viruses have a means to counteract the restriction has not been determined.

**Results:**

We show that SAMHD1 restricts a wide array of divergent retroviruses, including the alpha, beta and gamma classes. Murine leukemia virus was restricted by SAMHD1 in macrophages yet removal of SAMHD1 did not alleviate the block to infection because of an additional block to viral nuclear import. Prototype foamy virus (PFV) and Human T cell leukemia virus type I (HTLV-1) were the only retroviruses tested that were not restricted by SAMHD1. PFV reverse transcribes predominantly prior to entry and thus is unaffected by the dNTP level in the target cell. It is possible that HTLV-1 has a mechanism to render the virus resistant to SAMHD1-mediated restriction.

**Conclusion:**

The results suggest that SAMHD1 has broad anti-retroviral activity against which most viruses have not found an escape.

## Background

SAMHD1 is a recently identified component of the innate immune system that restricts the replication of HIV-1 in myeloid cells [1,2]. In HIV-2 and isolates of SIV from macaques (SIVmac), the SAMHD1-mediated restriction is counteracted by the Vpx accessory protein [3,4] whereas in viruses such as SIVagm, the restriction is counteracted by the related Vpr accessory protein. HIV-1 does not encode Vpx and its Vpr does not counteract SAMHD1 [1]. As a result, the virus replicates inefficiently in myeloid cells. U937 cells that are stably transduced with SAMHD1 lentiviral expression vector and differentiated with phorbol myristic acid (PMA) are resistant to HIV-1 and Δ*vpx* SIV while siRNA knock-down of SAMHD1 in primary monocyte-derived macrophages (MDM) increases their susceptibility to HIV-1 [1,2]. Aicardi-Goutieres Syndrome, a rare early onset neurological disease, is in some cases caused by polymorphisms in the *samhd1* gene that inactivate the enzyme. As a result of the absence of SAMHD1 the MDM of such patients support high levels of HIV-1 replication upon *in vitro* infection [5].

SAMHD1 belongs to a family of nucleases and phosphohydrolases that are distinguished by the presence of an HD domain [6]. *E*. *coli* produced recombinant SAMHD1 is a dGTP-stimulated triphosphohydrolase that removes the triphosphate from deoxynucleotide triphosphates (dNTPs) in a single step, converting dNTPs to nucleosides (dNs) [7,8]. SAMHD1 was first identified as a restriction factor in studies to identify myeloid host proteins that coimmunoprecipitate with Vpx [1,2]. In lentiviral-vector transduced U937 cells, SAMHD1 diminishes the pool of intracellular dNTPs, decreasing their concentration to a level below that required to support reverse transcription [9]. As a result, full-length reverse transcripts are not generated and the infection is blocked. In actively dividing cells, SAMHD1 has little effect on the dNTP pool [9].

The lentiviral Vpx accessory protein is a small, nuclear protein that is packaged into the virion during virus assembly. The packaging is mediated by an interaction with an amino acid motif located in p6 of the Gag precursor polyprotein Pr55^gag^[10]. The presence of Vpx in the virion allows it to act post-entry, prior to the synthesis of new virus protein. Upon release from the virion, Vpx is thought to form a complex with the CRL4A E3 ubiquitin ligase [11-13] that induces the ubiquitination and proteasomal degradation of SAMHD1. The decreased abundance of SAMHD1 restores the dNTP pool and releases the block to reverse transcription [9].

HIV-1 infection is sensitive to restriction by SAMHD1 yet the viral genome does not encode a Vpx protein. Introduction of Vpx into MDM or monocyte derived dendritic cells (MDDC) by pretreatment of the cells with Vpx-containing virus-like particles (VLP) increases the susceptibility of the cells to infection by HIV-1 and HIV-1-based lentiviral vectors [14-16]. Vpx-mediated enhancement has also been shown for feline immunodeficiency virus (FIV) infection and murine leukemia virus (MLV) reverse transcription [15]. HIV-1 in which the SIV Vpx packaging motif was engineered into Gag p6 was shown to package Vpx provided *in trans*. Vpx-containing HIV-1 was found to be nearly 100-fold more infectious on DC than control HIV-1 [17]. Vpx does not appear to have an effect on the ability of HIV-1 to infect activated CD4+ T cells [18].

Nucleotide pool depletion may serve as a host defense to protect against a variety of different viruses that are dependent upon the dNTP pool of the target cell. This mechanism might be particularly active against viruses that lack Vpx or a Vpx-like function, as it is the case for many lentiviruses and retrovirus families. To determine which retroviruses are susceptible to SAMHD1-mediated restriction, we tested a panel of retroviruses for their sensitivity to restriction by SAMHD1. We found SAMHD1 restricted all except for human T cell leukemia virus type 1 (HTLV-1) and the prototype foamy virus (PFV). PFV completes reverse transcription predominantly prior to virus entry, accounting for its ability to escape SAMHD1-mediated restriction.

## Results and discussion

To determine the sensitivity of diverse retroviruses to SAMHD1-mediated restriction, we used an assay in which SAMHD1 expressing cells were incubated with Vpx-containing VLP and then infected with reporter virus. This approach allowed us to analyze the effect of Vpx on viruses that do not themselves encode or package the protein. For the assay, we generated VSV-G pseudotyped, Vpx-containing and control VLP by transfection of 293 T cells. The VLP were normalized and then added to primary MDM and to PMA-differentiated THP-1 cells. THP-1 is a monocytic cell line that expresses endogenous SAMHD1 and that in the undifferentiated state is permissive to HIV-1 but upon differentiation with phorbol ester becomes restricted to infection [16,18]. After addition of VLP, the cells were infected with single-cycle reporter viruses and three days later the infection was quantified by luciferase assay or flow cytometry in the case of GFP and RFP reporter viruses.

**Figure 1:**
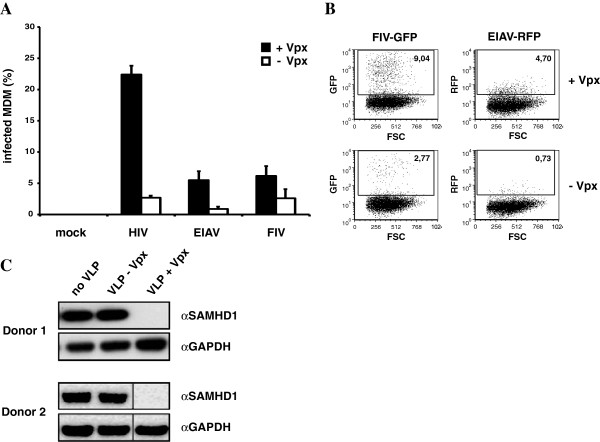
**SAMHD1 restricts lentiviral infection in myeloid cells.** (**A**) Vpx enhances lentiviral infection in MDM. MDM were preincubated for 2 h with Vpx-containing (+Vpx) or control VLP (−Vpx) and either not infected (mock) or infected with VSV-G-pseudotyped HIV-GFP, FIV-GFP, or EIAV-RFP reporter virus. Reporter virus infection was quantified three days later by flow cytometry. The percentage of infected cells is shown as average of duplicate infections. (**B**) FACS plot of one donor MDM infected as described in (**A**). (**C**) MDDC were incubated with Vpx-containing VLP, control VLP or no VLP for 24. Cell lysates were prepared and analyzed on an immunoblot probed with anti-SAMHD1 MAb or anti-GAPDH MAb. The results shown are representative of results obtained in three independent experiments.

Equine infectious anemia virus (EIAV) and FIV do not encode Vpx. EIAV encodes accessory proteins of unknown function, S2 and Ttm. Both proteins are dispensable for EIAV replication *in vitro* and are not virion-packaged and thus unlikely to have Vpx-like activity [19,20]. FIV encodes Vif and OrfA accessory proteins, which were not encoded by our reporter virus. FIV Vif serves to counteract APOBEC3 proteins [21,22] and OrfA acts as a transactivator analogous to HIV-1 Tat [23,24]. Neither is virion-packaged and thus not likely to serve as a Vpx homologue. To test the susceptibility of these viruses to SAMHD1-mediated restriction, we used the VLP assay with EIAV and FIV reporter viruses and an HIV-1 reporter virus control. The results showed that HIV-1 was sensitive to SAMHD1-mediated restriction as reflected in the nearly 10-fold boost to infectivity provided by Vpx (Figure 1A). EIAV was similarly sensitive to SAMHD1-mediated restriction, showing a 7-fold increase in response to Vpx-containing VLP, albeit at lower virus titer (Figure 1A, 1B). FIV was similarly responsive to Vpx. The enhancement of infection by Vpx-containing VLP in myeloid cells, like MDM or MDDC, correlated with the ability of Vpx to degrade endogenous SAMHD1 in these cells (Figure 1C).

**Figure 2:**
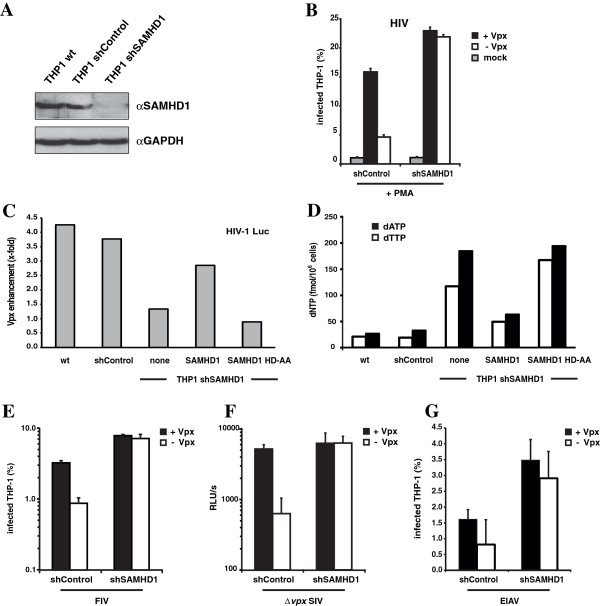
**SAMHD1 restricts HIV-1, FIV, SIV and EIAV infection in THP-1 cells.** (**A**) Differentiated THP-1 shSAMHD1, shControl or THP-1 wild-type cell lysates were analyzed on an immunoblot probed with anti-SAMHD1 MAb or anti-GAPDH MAb. (**B**) THP-1 shSAMHD1 and control cells were differentiated with PMA, preincubated with VLP and infected with HIV-GFP reporter virus at a MOI = 1. (**C**) THP-1 control or shSAMHD1 cells transduced with a lentiviral vector coding for shRNA-resistant SAMHD1, the catalytic-inactive mutant SAMHD1 HD-AA or empty vector (none), were differentiated then pretreated with Vpx-containing or control VLP and infected with HIV-luciferase reporter virus. Luciferase activity was measured three days postinfection and is shown as fold enhancement by Vpx-VLP treatment. (**D**) Quantification of dATP and dTTP nucleotide levels in differentiated SAMHD1 knock-down and control THP-1 cell lines. In (**E**), (**F**) and (**G**) Control or SAMHD1 knock-down THP-1 cells were differentiated with PMA, preincubated with Vpx-containing (+Vpx) or control VLP (−Vpx), and infected with (**E**) FIV-GFP, (**F**) with SIV-ΔVpx-luc, or (**G**) EIAV-RFP reporter virus. Reporter virus infection was quantified three days later by flow cytometry or luciferase assay. The data shown are the average of triplicate infections with error bars indicating the standard deviation.

To confirm these findings we generated THP-1 cell lines in which SAMHD1 expression was knocked-down using lentiviral vector-encoded shRNA and a control cell line transduced with a scrambled shRNA vector. The efficiency of the SAMHD1 knock-down was confirmed by analyzing endogenous protein levels in shControl and shSAMHD1 cells by western blot analysis (Figure 2A). Next, we preincubated the PMA-differentiated cells with Vpx-containing and control VLP and infected them with HIV-GFP. We found that the infection was restricted in control cells and relieved by Vpx-containing VLP (Figure 2B). In contrast, there was no restriction in knock-down cells and the Vpx-VLP had no effect. SAMHD1 knock-down was somewhat more effective than Vpx-VLP in relieving the restriction suggesting that virion-packaged Vpx does not fully counteract SAMHD1. To confirm that SAMHD1 is mediating the Vpx phenotype in THP-1 cells we reconstituted the knock-down THP-1 cells with a lentiviral vector encoding either shRNA-resistant wt SAMHD1 or a catalytically inactive mutant, SAMHD1 HD-AA, in which the amino acids Aspartate and Histidine at position 206–207 were replaced with Alanine (Figure 2C). We found that knock-down cells reconstituted with wt SAMHD1, but not with SAMHD1 HD-AA, restricted HIV-1 reporter virus infection again and that this restriction was counteracted by Vpx-VLP. This gain of function was accompanied with reduced dNTP levels in SAMHD1 cells, in sharp contrast to SAMHD1 HD-AA cells, indicating that the restriction in THP-1 cells is mediated by the nucleotide hydrolase activity of SAMHD1 (Figure 2D). To determine whether the restriction to FIV, EIAV and SIV was also caused by SAMHD1, we infected shControl and shSAMHD1 THP-1 cells with the respective reporter viruses (Figure 2E-G). The results showed that the SAMHD1 knock-down relieved the block to infection for all viruses and that Vpx-containing VLP had no additional effect (Figure 2E-G). We concluded that SAMHD1 is the host factor that restricts the infection of the lentiviruses HIV-1, SIV, EIAV and FIV in myeloid cells.

**Figure 3:**
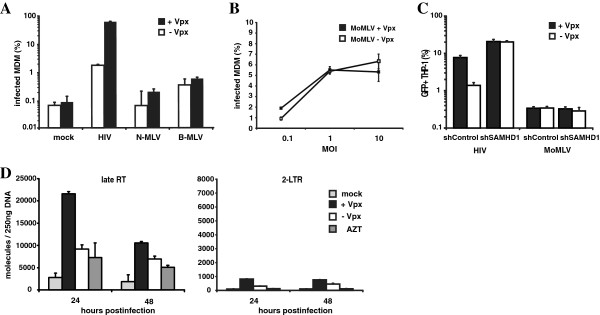
**Vpx relieves SAMHD1-mediated restriction of MLV RT but does not allow for productive infection of non-dividing cells.** (**A**) MDM were preincubated with Vpx-containing (+Vpx) or control VLP (−Vpx). The cells were then incubated with control supernatant (mock) or infected with VSV-G pseudotyped HIV-GFP, N-tropic MLV-GFP (N-MLV), or B-tropic MLV (B-MLV) at a MOI = 1 or (**B**) with increasing amounts of NB-tropic MLV-GFP (NB-MLV). (**C**) THP-1 cells that express shSAMHD1 (shSAMHD1) or control shRNA (shControl) were differentiated with PMA, preincubated with VLP and then infected with HIV-GFP or MLV-GFP at MOI = 1. (**D**) MDM were incubated with Vpx-containing (+Vpx) or control VLP (−Vpx). After 2 h, the cells were infected with control supernatant (mock) or with NB-tropic MLV reporter virus. Cellular DNA was isolated 24 and 48 h postinfection and used as a template in qPCR to amplify late reverse transcriptase products and 2-LTR circles. AZT was added to one well treated with Vpx-containing VLP prior to infection to control for contaminating plasmid. The data are presented as the average of triplicates with error bars indicating the standard deviation. Reporter gene expression in the infected cells was analyzed three days postinfection by flow cytometry. Similar results were obtained in three independent experiments with different donors.

Gamma retroviruses, such as MLV do not encode auxiliary genes and do not infect nondividing cells. To determine whether this class of retrovirus is sensitive to SAMHD1-mediated restriction, we tested different strains of MLV reporter virus in the VLP assay. Different strains of MLV were used for infection to exclude a potential influence of other restriction factors, like human TRIM5α on N-MLV infection. In this assay, Vpx-containing VLP increased the infectivity of HIV-1 by 35-fold as compared to control VLP on MDM (Figure 3A). In contrast, N- and B-tropic MLV were poorly infectious on MDM (<1% infected cells) and there was only a minor effect of Vpx-containing VLP. To determine whether the block to MLV infection could be saturated at higher MOI, we infected the cells with a third strain, NB-tropic MLV, over a range of MOI. At high MOI, 5% of the cells became infected (Figure 3B) but there was no significant effect of Vpx-containing VLP. The source of the 5% infection is not clear, but the lack of effect of Vpx suggests that it is the result of a small number of contaminating lymphocytes. Knock-down of SAMHD1 in differentiated THP-1 cells also did not relieve the block to MLV infection (Figure 3C).

Although Vpx did not relieve the block to MLV, it remained possible that it had an effect on the ability of the virus to reverse transcribe. To determine whether SAMHD1 affects the reverse transcription of MLV in MDM, we treated the cells with Vpx-containing VLP, infected with NB-tropic MLV and then quantified the late reverse transcripts and 2-LTR circles, a marker of nuclear import. We found that Vpx-containing VLP significantly enhanced the number of late reverse transcripts (Figure 3D). At 24 h post-infection, the late reverse transcripts increase about two-fold; however, if the background is subtracted the enhancement is about 10-fold. The background in this analysis is defined by the number of reverse transcripts measured in AZT-treated cells and probably results from intravirion reverse transcripts. By 48 h, the number of late reverse transcripts decreased, presumably reflecting degradation of the viral DNA in the cytoplasm. The number of 2-LTR circles remained low at both 24 and 48 h. Thus, SAMHD1 restricts MLV by blocking its reverse transcription yet removal of the block by Vpx does not allow for productive infection because the virus remains unable to penetrate the nucleus. This finding showed that MLV infection of MDM is restricted at two levels: reverse transcription and nuclear import.

**Figure 4:**
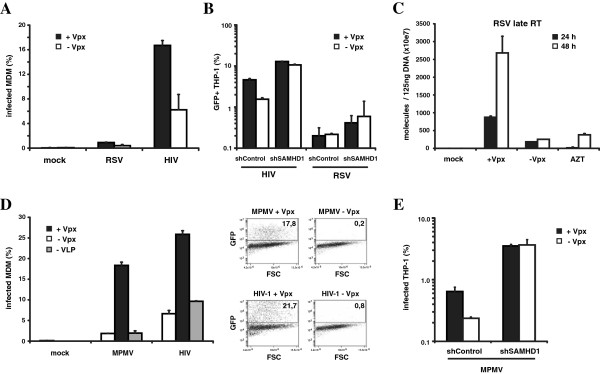
**SAMHD1 restricts MPMV and RSV.** (**A**) MDM or (**B**) PMA-treated THP-1 shControl and THP-1 shSAMHD1 cells were preincubated with Vpx-containing (+Vpx) or control VLP (−Vpx) and infected with control supernatant (mock) or with RSV-GFP or HIV-GFP reporter viruses at a MOI = 1. Reporter gene expression in infected cells was analyzed three days postinfection by flow cytometry. (**C**) MDM were incubated with Vpx-containing (+Vpx) or control VLP (−Vpx) and then infected with control supernatant (mock) or with RSV reporter virus. Cellular DNA was purified 24 and 48 h postinfection and used to quantify late reverse transcription products by qPCR. AZT was added to one well treated with Vpx-containing VLP prior to infection (AZT). (**D**) MDM were preincubated for 2 h with Vpx-containing (+Vpx) or control VLP (−Vpx) and not infected (mock) or infected with MPMV-GFP or HIV-GFP at a MOI = 1. The data are shown on the left as a histogram and on the right as a FACS plot. (**E**) MPMV infection as in (**D**) but using PMA-differentiated THP-1 shControl and THP-1 shSAMHD1 cells. Reporter gene expression was analyzed three days postinfection by flow cytometry. Reporter gene expression was analyzed as shown for HIV-GFP and MLV-GFP infection of MDM blotted in (**D**). The data presented are the average of triplicates with error bars indicating the standard deviation. The results of one of three independent experiments are shown.

Alpha retroviruses such as Rous Sarcoma Virus (RSV) do not encode accessory proteins. Analysis of the sensitivity of RSV to SAMHD1-mediated restriction showed that the virus failed to infect MDM and that the block was not relieved by Vpx-containing VLP (Figure 4A). RSV also did not infect differentiated THP-1 SAMHD1 knock-down cells (Figure 4B). Analysis of the number of reverse transcripts showed that, as for MLV, Vpx-containing VLP relieved the block to reverse transcription in MDM (Figure 4C).

The beta retrovirus Mason Pfizer Monkey virus (MPMV) assembles in the cytoplasm and does not encode accessory proteins. An analysis of its sensitivity to SAMHD1-mediated restriction shows that Vpx-containing VLP enhanced its ability to infect MDM (Figure 4D) and that knock-down of SAMHD1 in differentiated THP-1 relieved the block to infection (Figure 4E). Thus, MPMV is sensitive to the SAMHD1 restriction but differs from MLV and RSV with respect to its ability to infect nondividing cells.

**Figure 5:**
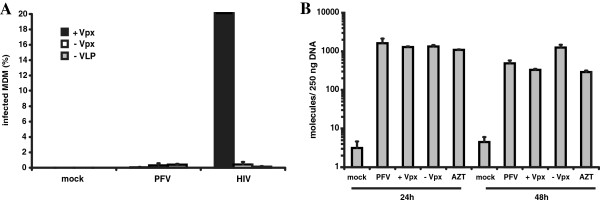
**Vpx does not enhance PFV infection of MDM.** (**A**) MDM were preincubated with Vpx-containing (+Vpx) or control VLP (−Vpx) or medium and were not infected (mock) or infected with PFV-GFP or HIV-GFP in triplicate at MOI = 1. Three days postinfection, the infected cells were quantified by flow cytometry. One out of three independent experiments is shown. (**B**) MDM were incubated with Vpx-containing or control VLP or medium (PFV) and then not infected (mock) or infected with PFV reporter virus at an MOI =1. After 24 and 48 h, cellular DNA was isolated and PFV DNA was quantified by qPCR. AZT was added to one well treated with Vpx-containing VLP prior to infection to control for plasmid contamination (AZT). The data are the average of triplicates with error bars indicating the standard deviation. Similar results were obtained with cells from two different donors.

Foamy viruses do not encode Vpx but have the accessory proteins Tas and Bet [25]. The viruses do not infect nondividing cells [26]. An unusual feature of these viruses is that they reverse transcribe late in the replication cycle [27]. As a result, many virions contain nearly completed viral genomic DNA. To determine the sensitivity of this virus to SAMHD1-mediated restriction, we used the VLP assay with PFV-GFP reporter virus. Infectivity of control HIV-GFP was enhanced 20-fold by Vpx-containing VLP while PFV infection was unaffected (Figure 5A). Quantitative PCR analysis of the number of reverse transcripts in the MDM showed no effect of Vpx (Figure 5B). Addition of AZT to the target cells had no effect on the number of reverse transcripts consistent with the ability of PFV to complete reverse transcription prior to infection.

**Figure 6:**
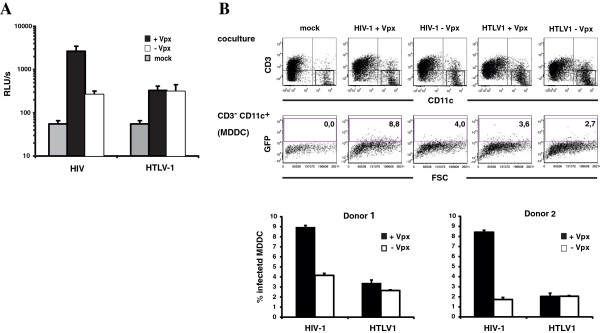
**Vpx does not enhance HTLV-1 transmission to MDM or MDDC.** (**A**) Jurkat cells were cotransfected with an antisense-intron-containing HTLV-1 or HIV-1 luciferase reporter plasmid, the respective packaging plasmid and pVSV-G. After 24 h, the transfected Jurkat cells were cocultured at a ratio of 2:1 with MDM that had been preincubated for 2 h with 25 ng p27 of Vpx-containing (+Vpx) or control VLP (−Vpx). After 48 h, the Jurkat cells were removed and adherent MDM were lysed and the luciferase activity was determined in quadruplicates. Background luciferase activity was determined by transfection of Jurkat cells with control plasmids (mock). The data are expressed as relative light units (RLU). One of four independent experiments is shown. (**B**) Jurkat cells were cotransfected with HTLV-1 or HIV-1 GFP reporter plasmids, the respective packaging plasmid and pVSV-G. After 24 h, the transfected Jurkat cells were cocultured at a ratio of 2:1 with MDDC that had been pretreated with 25 ng p27 of Vpx-containing (+Vpx) or control VLP (−Vpx). After 48 h, the cells were analyzed by flow cytometry to detect transmission of HIV-1 GFP and HTLV-1 GFP from the Jurkat cells to the MDDC by staining with anti-CD3-PECy5 to detect the Jurkat cells and anti-CD11c-APC to detect the MDDC. The infected MDDC were defined as the CD3-/CD11c + population. The data are the average of triplicate infections with error bars to indicate the standard deviation. One of three independent experiments is shown.

HTLV-1 primarily infects T cells *in vivo* but at least *in vitro* can infect DC and MDM [28]. Cell-free HTLV-1 particles are poorly infectious suggesting that the virus spreads primarily by cell-to-cell contact. To determine the sensitivity of HTLV-1 to SAMHD1-mediated restriction, we used a coculture assay in which Jurkat T cells were cotransfected with plasmids encoding the structural proteins, VSV-G and a reporter virus plasmid that contained a luciferase gene in the antisense orientation interrupted by an intron in the sense orientation [29]. This configuration allows the reporter gene to be expressed in the infected cells but not in the transfected producer cells. For the analysis, we treated MDM with Vpx-containing or control VLP and then added virus-producing Jurkat cells. After 48 h, we removed the Jurkat cells by washing and measured luciferase activity in the adherent cells. We found that there was no significant difference in the amount of luciferase activity between MDM pretreated with Vpx-containing and control VLP for HTLV-1 (Figure 6A). Control HIV-1 infection was enhanced 8-fold by Vpx-containing compared to control VLP. To exclude the possibility of contaminating Jurkat cells in our MDM luciferase readout we repeated the coculture assay described above with Jurkat cells transfected with GFP reporter constructs and MDDC (Figure 6B). After 48 h of coculture, the cells were probed with anti-CD3 and anti-CD11c antibodies and analyzed by flow cytometry. We determined infectivity (GFP + cells) in CD3-negative CD11c-positive MDDC and found that, in contrast to HIV, the transmission of HTLV-1 from Jurkat T cells to MDDC was not enhanced by Vpx (Figure 6B).

## Conclusions

For most retroviruses, reverse transcription requires a preexisting pool of dNTPs in the target cell. Thus, nucleotide pool depletion by SAMHD1 could be a general mechanism by which the cell restricts the replication of various genera of retroviruses. Our findings show that alpha, beta and gamma retroviruses were indeed restricted by SAMHD1 in myeloid cells. These findings are consistent with those of White *et al*. who recently reported that bovine immunodeficiency virus and EIAV are sensitive to SAMHD1 [30]. Unlike HIV-2 and SIV, these viruses do not appear to have a mechanism to escape the restriction. Two of the viruses tested were relatively insensitive to SAMHD1-mediated restriction. The foamy virus PFV completes reverse transcription predominantly prior to entry and therefore does not utilize the target cell pool of dNTPs to synthesize its genome. The resistance of PFV to SAMHD1-mediated restriction did not allow the virus to productively infect MDM, suggesting that the virus cannot penetrate the nuclear membrane of non-mitotic cells. HTLV-1 also appeared to be unaffected by SAMHD1. This may be because the virus encodes an accessory protein with Vpx-like activity. HTLV-1 encodes p8, p12, p13, p30 and HBZ accessory proteins, one of which might have such a function [31]. However, in experiments in which HTLV-1-based VLPs were added to target cells (Additional file 1: Figure S1) and in which cells were transfected with HTLV-1 DNA (Additional file 2: Figure S2), we did not detect degradation of SAMHD1 under conditions in which SIV Vpx showed a clear effect. Thus, HTLV-1 may have another means of avoiding SAMHD1-mediated restriction.

MLV was restricted by SAMHD1 but Vpx did not allow the virus to infect the MDM. Although MLV did not replicate in the MDM, Vpx relieved the block to reverse transcription. In spite of the synthesis of viral reverse transcripts upon Vpx-induced degradation of SAMHD1, the lack of 2-LTR DNA circles suggests that the virus was unable to penetrate the nucleus of the MDM. At high MOI about 5% of the cells became infected; however, this percentage was unaffected by Vpx and thus unrelated to SAMHD1 and most likely the result of low level contamination of the MDM population with activated T cells. Our findings are inconsistent with those of Kaushik *et al*. who found that Vpx allowed productive infection of MDM by MLV [32]. The source of this discrepancy is not clear.

Interestingly, once the SAMHD1-mediated block was relieved by Vpx, MPMV infected MDM. This finding demonstrates that the ability to navigate the nuclear pore in nondividing cells is not limited to lentiviruses and furthermore, that a retrovirus that lacks accessory proteins has found a mechanism to access the nucleus in nondividing cells. This feature does not allow MPMV to infect myeloid cells, as the virus was still restricted by SAMHD1, but could allow the virus to infect nondividing cell-types that have a sufficient pool of dNTPs.

The presence of Vpx in HIV-2 and SIV demonstrates that it is possible for retroviruses to escape SAMHD1-mediated restriction. Thus, it is somewhat surprising that most do not encode Vpx and have no mechanism to counteract SAMHD1. The lack of such a mechanism may suggest that *in vivo*, most retroviruses do not depend upon infection of myeloid cells as a means of spreading. It has been suggested that in fact, it may be advantageous for viruses to avoid infecting myeloid cells because of their role in stimulating innate immune defenses and in presenting processed peptide antigens to T cells [33]. Alternatively, in species other than primates, SAMHD1 may not play a similar role in depleting the dNTP pool. Although nucleotide pool depletion is an antiviral mechanism that is limited to nondividing cells that do not need a high level of dNTPs, it is a mechanism that affects a broad range of retroviruses and may extend to other pathogens that rely upon DNA synthesis to replicate their genomes.

## Methods

### Cells and cell culture

293 T cells were cultured in Dulbecco’s modified Eagle medium / 10% fetal bovine serum (FBS). Jurkat and THP-1 cells were cultured in RPMI 1640 / 10% FBS. For THP-1 cells containing a shRNA-encoding lentiviral vector, 500 ng/ml puromycin was added to the medium. THP-1 cells were differentiated by culturing for three days with 50 nM phorbol 12-myristate 13-acetate (PMA). Monocytes were purified from healthy donor peripheral blood mononuclear cells (PBMC) by adherence to plastic or positive selection on anti-CD14-coated magnetic beads (Miltenyi Biotech) and were typically >98% CD14+. Monocytes were differentiated to MDM by culturing for 5 to 7 days in medium containing 50 ng/ml of granulocyte-macrophage colony-stimulating factor (GM-CSF). Monocytes were differentiated to MDDC by culturing for four days in RPMI 1640 supplemented with 5% pooled human serum, 1 mM HEPES, gentamycin, 50 ng/ml GM-CSF (Invitrogen) and 300 U/ml Interleukin-4 (IL-4, R&D systems)

### Plasmids

Lentiviral vectors coding for short hairpin RNA targeting SAMHD1 (shSAMHD1) or a scrambled shRNA (shControl) and the vector for overexpression of shRNA-resistant wt SAMHD1 (pLenti-SAMHD1-shRNA^r^-*neo*) have been described previously [34]. The shRNA-resistant, catalytically inactive mutant SAMHD1 HD-AA was cloned into pLenti-*neo* (Addgene) using a similar strategy. Briefly, the HD206-207AA mutation was introduced in shRNA-resistant SAMHD1 by overlapping PCR mutagenesis. The amplicon was digested with BamHI and XhoI and cloned back into pLenti-*neo* via BamHI- and SalI. The resulting construct was confirmed by nucleotide sequencing. The pSIVmac-luc-E^-^ reporter constructs and the plasmids used to generate SIV VLP, pSIV3+ and pSIV3 + X^-^, have been previously described [14,16]. All SIV-derived proviral plasmids are based on SIVmac239 with the exception of pSIV3+ and pSIV3 + X^-^, which are based on SIVmac251. The env-deficient HIV-1 reporter plasmids pNL43-E^-^CMVGFP expressing GFP under control of a CMV promoter and pNL-Luc3-E^-^ expressing the luciferase reporter gene have been described previously [34,35]. The EGFP-expressing prototype foamy virus (PFV) construct, pczDWP001, and the plasmid encoding PFV Env, pczHFVenvEM02, were described previously [36,37]. The reporter virus plasmids for FIV (pFP93 and pGINSIN) [38,39], EIAV (pONY3.2), RSV (pAlpha.SF.EGFP.WPRE and pcDNA.alpha.gag/pol.CO) [40], and MPMV (pSARM-EGFP) [41,42] have been previously described. pEIAV-RFP was a kind gift of Taichiro Takemura and Vineet KewalRamani and was derived from p6.1G3CeGFPW [43]. The *env*-deleted HTLV-1 packaging plasmid pCMVHT1-ΔX, the intron-containing HTLV-1 luciferase reporter plasmid pCRU5HT1-inLuc, the *env*-deficient HIV-1 packaging plasmid pCMV.PA-HIV and the intron-containing HIV-1 luciferase reporter plasmid, pUCHRinLuc have been described by Mazurov *et al*. [29].

### Virus preparation and infections

Reporter viruses were produced in 293 T cells cotransfected with *env*-deficient reporter virus plasmids and pVSV-G vesicular stomatitis virus glycoprotein expression plasmid using Lipofectamine 2000 (Invitrogen) or by calcium phosphate coprecipitation. SIVmac239-ΔVpx luciferase reporter virus (SIV-ΔVpx-luc) was generated as described previously [44]. HIV-1-GFP was generated by cotransfection of pNL43-E^-^CMVGFP and pVSV-G at a mass ratio of 4:1. HIV-1-Luciferase (HIV-luc) reporter virus was generated by cotransfection of pNL-Luc3-E^-^ and pVSV-G at a mass ratio of 5:1. The EIAV reporter virus was produced by transfection of pONY3.2, pEIAV-RFP, and pVSV-G at a ratio of 2:2:1. FIV-ΔVif-ΔOrfA-ΔEnv-GFP reporter virus was produced by transfecting pFP93, pGINSIN, and pVSV-G at a mass ratio of 3:3:1. MLV-GFP reporter virus (NB-tropic) was produced by transfection with pMX-GFP, pHIT-60, and pVSV-G at a mass ratio of 4:2:1. For the production of N-tropic and B-tropic MLV-GFP, pHIT60 (NB-tropic MLV gagpol) was replaced with pN-MLVgagpol or pB-MLVgagpol in the transfection mix. RSV-EGFP reporter virus was produced by cotransfection of the retroviral vector pAlpha.SF.EGFP.WPRE, the packaging plasmid pcDNA.alpha.gag/pol.CO, and pVSV-G at a ratio of 2:2:1. MPMV-GFP reporter virus was generated by transfection with pSARM-EGFP plasmid and pVSV-G at a mass ratio of 4:1. To generate PFV-GFP reporter virus the plasmids pczDWP001 and pczHFVenvEM02 were cotransfected at a mass ratio of 1:1. To produce VLP, 293 T cells were cotransfected with pSIV3+, or pSIV3 + X-, and pVSV-G at a mass ratio of 4:1. After 6 h, the culture medium was replaced. Supernatants were harvested 48 h posttransfection, passed through 0.4-μm-pore size filters, aliquotted and frozen at −80°C.

SIV and HIV luciferase reporter viruses were normalized on 293 T cells by luciferase assay according to the manufacturer’s instructions (Promega). For this, 1 X 10^4^ 293 T cells were infected with 10, 30, and 50 μl of SIV-luc reporter virus. After 72 h, the infected cells were lysed and luciferase activity was measured. The viruses had an average activity of 0.5 - 2.0 × 10^6^ counts per second (cps)/μl. GFP and RFP reporter viruses were titered on 293 T cells. Infected cells were analyzed 72 h postinfection by flow cytometry. VLP were normalized for SIV Gag p27 content measured by ELISA (Innogenetics).

In reporter virus infection assays, MDM and THP-1 cells (2.5 × 10^5^) were spin-infected in 1 ml RPMI for 2 h at 500 × g with GFP or RFP reporter viruses at a MOI = 1 or with 4 × 10^5^ cps of luciferase reporter viruses unless otherwise indicated. Two hours prior to infection, the cells were incubated with supernatant containing 25 ng SIV-VLP in a volume of 0.5 ml RPMI. After 6 h, the medium was replaced. The number of infected cells was determined after three days for THP-1 or four days for MDM by flow cytometry or luciferase assay.

### Quantitative real-time PCR (qPCR)

MDM were infected at a MOI = 1 with virus stocks treated for 1 h with 50 U of Benzonase (Invitrogen)/ml to remove contaminating plasmid DNA. 25 μM AZT was added to one well 14 h prior to infection to control for residual plasmid DNA. After 24 h and 48 h, DNA was isolated using a QIA Amp DNA Kit (Qiagen). Reverse transcripts in 250 ng of total DNA were quantitated by qPCR with an ABI Prism 7300 (Applied Biosystems) and SYBR green reagent (Fermentas). The primer pairs used to detect RSV late reverse transcription products were 5^′^-lateRT-RSV (5^′^-TGTGCACCTGGGTTGATGGC) and 3^′^-lateRT-RSV (5^′^-TGGAGACAGGATCGCCACGC); for late MLV, 5^′^-MLV-lateRT (5^′^-CGTCAGCGGGGGTCTTTC) and 3^′^ MLV lateRT (5^′^-CTGGGCAGGGGTCTCCCG); and for MLV 2LTR circles, 5^′^-MLV-2LTR (5^′^-CCTCTGAGTGATTGACTACCC) and 3^′^-MLV-2LTR (5^′^-CTTAAGCTAGCTTGCCAAACC). For PFV, the primers hybridized to a conserved part of the *pol* gene that was present in the PFV plasmid. The primers used were PFV-sense (5^′^-CTTCAACCTTTGCTGAATG), PFV-antisense (5^′^-TAATACAGGGCTATAGGTGT) and a TaqMan probe (5^′^-FAM-TTGGAATTCAGTACTCCTTATCACCC-BHQ1) [45]. Standard curves were generated using serially diluted proviral and 2-LTR plasmids.

### HTLV-1 and HIV-1 cell-to-cell transmission assay

Jurkat T cells (1 × 10^7^ cells) were electroporated in a volume of 0.8 ml RPMI with 27.5 μg of HIV (pUCHRinLuc) or HTLV-1 (pCRU5HT1-inLuc) intron-luciferase vectors, 17.5 μg of the HIV or HTLV *env*-deficient packaging plasmids and 5.0 μg of pVSV-G or empty vector using a Gene Pulser Xcell (BioRad). After 24 h, the cells were washed twice with PBS and 5 × 10^5^ Jurkat cells in 500 μl RPMI were added on top of 2.5 × 10^5^ MDM target cells in 1 ml RPMI. The MDM target cells were pretreated for 2 h with 25 ng p27 of Vpx-containing or control VLP. After 48 h of coculture, the cultures were washed three times with PBS to remove the Jurkat cells. The adherent MDM were then lysed and the luciferase reporter gene activity was determined.

### shRNA knockdown of SAMHD1 in THP-1 cells

Generation of THP-1 cells stably expressing control shRNA, SAMHD1-shRNA, or both SAMHD1-shRNA and shRNA-resistant SAMHD1 protein was described before [34]. Complementation of THP-1 cells with catalytically inactive SAMHD1 HD-AA was generated similarly. Briefly, THP-1 shSAMHD1 cells were transduced with pLenti-*neo* vector encoding the shRNA-resistant SAMHD1-HD206-7AA mutant and then selected with 1.0 μg/ml puromycin and 0.4 mg/ml G418.

### Immunoblot analysis

Cells were lysed in NP40 lysis buffer (50 mM HEPES, 150 mM KCl, 2 mM EDTA, 0.5% NP-40, Halt Protease Inhibitor). Lysates were quantified by Bradford assay (BioRad). 30 μg per sample were separated by SDS-PAGE, transferred to PVDF membranes and probed with an anti-SAMHD1 mouse MAb (Origene), or an anti-GAPDH mouse MAb (Ambion). The membranes were then probed with anti-mouse horseradish peroxidase (HRP)-labeled secondary antibody (goat, Sigma), and visualized using HRP substrate (Pierce) on an Odyssey imaging system (Li-Cor).

### Degradation assay

Degradation assays were performed as previously described [34]. Briefly, after four days of differentiation, MDDCs were seeded in a 24-well plate (4 × 10^5^ cells). The next day, the MDDCs were incubated with Vpx-containing or control VLP for 16 h. The cells were then lysed in NP40 lysis buffer. SAMHD1 was detected by immunoblot analysis as described above.

### dNTP quantification

THP-1 shControl, THP-1 shSAMHD1, or THP-1 shSAMHD1 cells complemented with shRNA-resistant SAMHD1 were differentiated with PMA for 20 h. Then 2 × 10^6^ cells were lysed in 65% methanol. The levels of dNTPs in the lysates were determined by single nucleotide extension assay as described previously [46].

## Abbreviations

RLU: Relative light units; MOI: Multiplicity of infection; PMA: Phorbol-12-myristate-13-acetate; AZT: Azidothymidine; MDM: Monocyte-derived macrophages; VLP: Virus-like particles; VSV-G: Vesicular stomatitis virus glycoprotein; HIV: Human immunodeficiency virus; SIV: Simian immunodeficiency virus; HTLV-1: Human T cell leukemia virus 1; PVF: Prototype foamy virus; FIV: Feline immunodeficiency virus; EIAV: Equine infectious anemia virus; MLV: Murine leukemia virus.

## Competing interests

The authors declared that they have no competing interest.

## Authors’ contributions

TG, TK, SW, EM and DL prepared viruses and tested them for sensitivity to SAMHD1-mediated restriction, NB prepared the SAMHD1 and knock-down cell lines, WD and BK analyzed the dNTP levels, HH performed the degradation assay, TG and NRL wrote the manuscript. All authors read and approved the final manuscript.

## Supplementary Material

Additional file 1 Figure S1 HTLV-1 does not induce SAMHD1 degradation in THP-1 cells. VSV-G pseudotyped HTLV-1 virions were produced by transfection of pCMVHT1-ΔX and pcVSV-G. A fraction of the viral supernatant was lysed in RIPA buffer and the transfected cells were lysed in NP40 buffer. **A**) SIV-VLP and HTLV virions were detected by immunoblot analysis of the producer cells and the viral supernatant lysates. **B**) SAMHD1 degradation by SIV and HTLV-1 was tested by incubation of PMA-differentiated THP-1 cells with an increasing amount of virus for 16h. Cells were lysed in NP40 buffer. Lysates were separated by SDS-PAGE and SAMHD1 was detected on an immunoblot. Click here for file

Additional file 2 Figure S2 HTLV-1 does not induce SAMHD1 degradation upon transfection in 293T cells. 293T cells were cotransfected with increasing amounts of plasmids coding for (**A**) HA-SAMHD1 or (**B**) Myc-SAMHD1, together with control vector or the indicated proviral construct. The plasmids pSIV3+ (SIVmac) and pCMVHT1-ΔX (HTLV-1) both encode the viral Gag, Pol and accessory proteins. After 48h, the cells were lysed in NP40 buffer. Lysates were separated by SDS-PAGE and SAMHD1 was detected on an immunoblot. Click here for file
