# Supplementary material for: Restriction of diverse retroviruses by SAMHD1
Source: Retrovirology. 2013 Mar 5;10:26. doi: 10.1186/1742-4690-10-26 (PMC3605129; doi:10.1186/1742-4690-10-26)
Supplement: Additional file 1 Figure S1 — HTLV-1 does not induce SAMHD1 degradation in THP-1 cells. VSV-G pseudotyped HTLV-1 virions were produced by transfection of pCMVHT1-ΔX and pcVSV-G. A fraction of the viral supernatant was lysed in RIPA buffer and the transfected cells were lysed in NP40 buffer. A) SIV-VLP and HTLV virions were detected by immunoblot analysis of the producer cells and the viral supernatant lysates. B) SAMHD1 degradation by SIV and HTLV-1 was tested by incubation of PMA-differentiated THP-1 cells with an increasing amount of virus for 16h. Cells were lysed in NP40 buffer. Lysates were separated by SDS-PAGE and SAMHD1 was detected on an immunoblot. [file 1742-4690-10-26-S1.pdf]

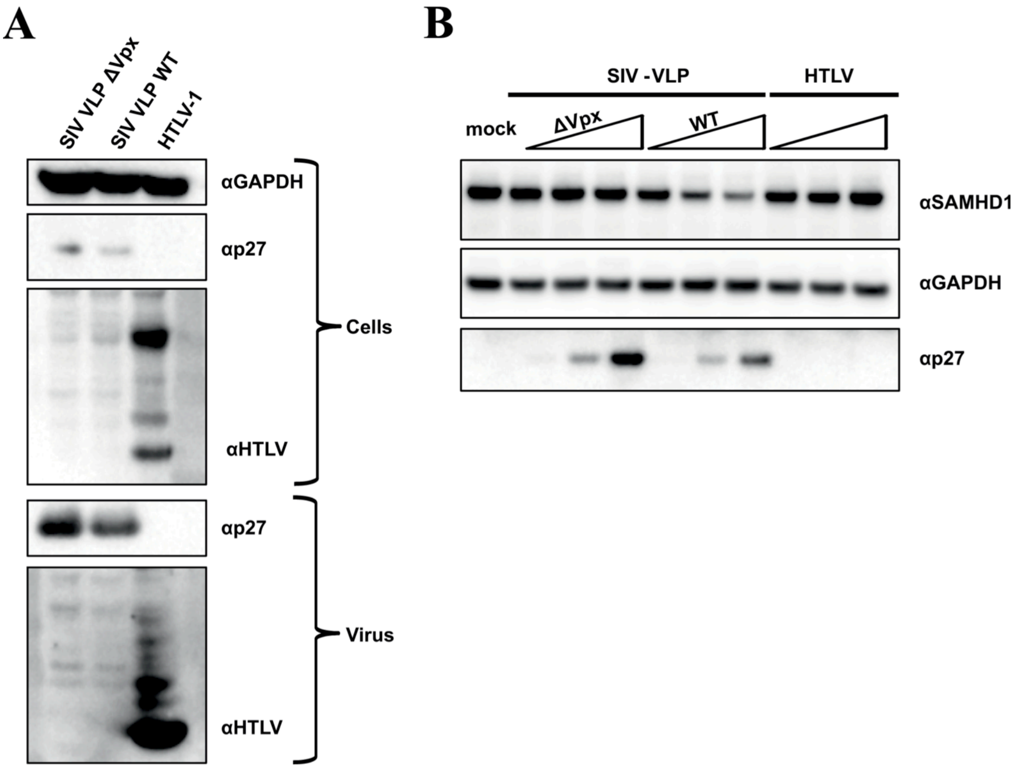


**Supplementary Figure 1. HTLV-1 does not induce SAMHD1 degradation.** VSV-G pseudotyped HTLV-1 virions were produced by transfection of pCMVHT-ΔEnv (Derse *et al*., J. Virol. 2001) and pcVSV-G. A fraction of the viral supernatant was lysed in RIPA buffer and the transfected cells were lysed in NP40 buffer. A) SIV-VLP and HTLV virions were detected by immunoblot analysis of the producer cells and the viral supernatant lysates. B) SAMHD1 degradation by SIV and HTLV was tested by incubation of PMA-differentiated THP1 cells with an increasing amount of virus for 16h. Cells were lysed in NP40 buffer. Lysates were separated by SDS-PAGE and SAMHD1 was detected on an immunoblot.
