# Supplementary material for: Restriction of diverse retroviruses by SAMHD1
Source: Retrovirology. 2013 Mar 5;10:26. doi: 10.1186/1742-4690-10-26 (PMC3605129; doi:10.1186/1742-4690-10-26)
Supplement: Additional file 2 Figure S2 — HTLV-1 does not induce SAMHD1 degradation upon transfection in 293T cells. 293T cells were cotransfected with increasing amounts of plasmids coding for (A) HA-SAMHD1 or (B) Myc-SAMHD1, together with control vector or the indicated proviral construct. The plasmids pSIV3+ (SIVmac) and pCMVHT1-ΔX (HTLV-1) both encode the viral Gag, Pol and accessory proteins. After 48h, the cells were lysed in NP40 buffer. Lysates were separated by SDS-PAGE and SAMHD1 was detected on an immunoblot. [file 1742-4690-10-26-S2.pdf]

A

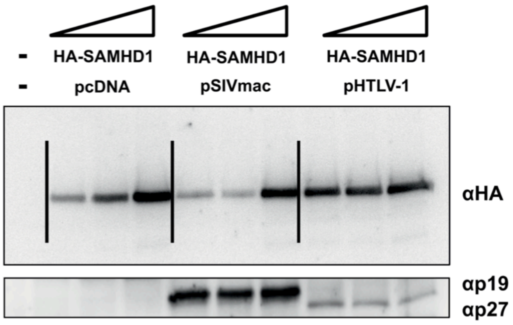

B

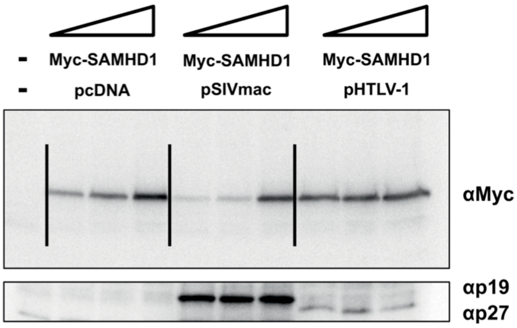

Supplementary Figure 2. HTLV-1 does not induces SAMHD1 degradation in 293T cells. 293T cells were cotransfected with increasing amounts of plasmids coding for (A) HA-SAMHD1 or (B) Myc-SAMHD1, together with control vector or the indicated proviral construct. The plasmids pSIV3+ (SIVmac) and HTLV-1-dX (HTLV-1) both encode the viral Gag, Pol and accessory proteins. After 48h, the cells were lysed in NP40 buffer. Lysates were separated by SDS-PAGE and SAMHD1 was detected on an immunoblot.
